# Direct mass spectrometry analysis of biological tissue for diagnosis of thyroid cancer using wooden-tip electrospray ionization

**DOI:** 10.3389/fchem.2023.1134948

**Published:** 2023-02-09

**Authors:** Dasheng Liu, Yuejian Shen, Dandan Di, Shenhui Cai, Xueyang Huang, Hongguo Lin, Yalan Huang, Jing Xue, Li Liu, Bin Hu

**Affiliations:** ^1^ Department of Vascular Thyroid Surgery, The Second Affiliated Hospital of Guangzhou University of Chinese Medicine, Guangzhou, China; ^2^ Hangzhou Linping Hospital of Traditional Chinese Medicine, Hangzhou, China; ^3^ Institute of Mass Spectrometry and Atmospheric Environment, Guangdong Provincial Engineering Research Center for On-Line Source Apportionment System of Air Pollution, Jinan University, Guangzhou, China; ^4^ Guangdong MS Institute of Scientific Instrument Innovation, Guangzhou, China; ^5^ Zhejiang Province Joint Key Laboratory of Aquatic Products Processing, Collaborative Innovation Center of Seafood Deep Processing, Institute of Seafood, Zhejiang Gongshang University, Hangzhou, China; ^6^ Health Management Center, The First Affiliated Hospital of Jinan University, Guangzhou, China

**Keywords:** thyroid cancer, tissue analysis, wooden tip, ambient mass spectrometry, electrospray ionization

## Abstract

Direct mass spectrometry (MS) analysis of human tissue at the molecular level could gain insight into biomarker discovery and disease diagnosis. Detecting metabolite profiles of tissue sample play an important role in understanding the pathological properties of disease development. Because the complex matrices in tissue samples, complicated and time-consuming sample preparation processes are usually required by conventional biological and clinical MS methods. Direct MS with ambient ionization technique is a new analytical strategy for direct sample analysis with little sample preparation, and has been proven to be a simple, rapid, and effective analytical tools for direct analysis of biological tissues. In this work, we applied a simple, low-cost, disposable wooden tip (WT) for loading tiny thyroid tissue, and then loading organic solvents to extract biomarkers under electrospray ionization (ESI) condition. Under such WT-ESI, the extract of thyroid was directly sprayed out from wooden tip to MS inlet. In this work, thyroid tissue from normal and cancer parts were analyzed by the established WT-ESI-MS, showing lipids were mainly detectable compounds in thyroid tissue. The MS data of lipids obtained from thyroid tissues were further analyzed with MS/MS experiment and multivariate variable analysis, and the biomarkers of thyroid cancer were also investigated.

## 1 Introduction

Thyroid cancer is the most common malignancy of the endocrine system that affects the thyroid gland, which is a small gland at the base of the neck that produces hormones (Wojakowska et al., 2015). There was a significant increase in the number of patients diagnosed with thyroid cancer over the past decades (Nguyen et al., 2015). Clinical diagnosis of thyroid cancer is mainly attributable to the developments and applications of modern imaging techniques, including ultrasounds, computed tomography, magnetic resonance imaging, positron emission tomography scans and others (Schneider and Chen, 2013; Nguyen et al., 2015). Bioanalysis of human tissue at a molecular level is an important task in human biology. Understanding molecular compositions of tissue allows us to monitor development and variation of human individuals, discover markers for disease diagnosis and gain insight into the mechanism of diseases. Molecular diagnostics have been developed by significant expansion in the understanding of the molecular basis of thyroid carcinogenesis (Nikiforov and Nikiforova, 2011), which could provide patients the personalized therapy. Various molecular methods have been developed for pathological diagnosis of thyroid cancer. Metabolic changes associated with diseased conditions could offer important insights into pathological mechanisms and help discovery of disease biomarkers (Sreekumar et al., 2009; Newgard, 2017). Metabolomics was thus developed for the characterization of metabolite changes in response to pathological changes (Levine and Puzio-Kuter, 2010).

Different analytical approaches, such as liquid chromatography (LC), gas chromatography (GC), mass spectrometry (MS), and nuclear magnetic resonance (NMR), are well accepted and widely used in metabolite studies (Griffin and Shockcor, 2004), many of which have been developed for metabolite studies of thyroid cancer (Wojakowska et al., 2015). Among these methods, MS is a rapid and sensitive tool for qualitative and quantitative analysis of various biological samples (Chughtai and Heeren, 2010). By use of these conventional MS-based analytical methods, typical sample pretreatment and chromatographic separation, which are time-consuming and complicated processes, are usually required prior to MS analysis. Therefore, these conventional MS-based analytical methods are limited to rapid analysis of metabolites of biological tissue. The development of ambient MS with ambient ionization techniques have greatly facilitated rapid analysis of biological tissues (Xiao et al., 2020). Direct analysis of tissues by ambient MS has been achieved mainly with various ambient ionization techniques including, desorption electrospray ionization (DESI), matrix-assisted laser desorption ionization (AP IR-MALDI), laser ablation electrospray ionization (LAESI) electrospray-assisted laser desorption/ionization (ELDI), air flow-assisted desorption electrospray ionization (AFADESI), and other ambient desorption/ionization (Shrestha et al., 2010; Tamura et al., 2019; Xiao et al., 2020; Huang et al., 2022). These techniques employ charged microdroplets and/or laser (with or without the assistance of a matrix) to desorb and ionize analytes on a tissue surface, and can be used for tissue imaging (Xiao et al., 2020; Huang et al., 2022). Ambient MS with paper spray ionization have been developed direct analysis of raw samples (Wang et al., 2011; Cai et al., 2021; Nguyen et al., 2022; Yang et al., 2022). Other ambient ESI techniques for direct MS analysis of biological tissue have been also developed using different solid substrates, e.g., paper (Wang et al., 2011), metal probe (Mandal et al., 2012), metal foil (So et al., 2019), tissue (Hu et al., 2012), wooden tip (Hu and Yao, 2018), and others (Hu and Yao, 2022). In tissue-ESI, analytes on a tissue surface are directly extracted by organic solvent and then sprayed out an ESI plume (Hu et al., 2012); while a tissue surface is loaded on a solid needle, and a trace biofluid adhering to the needle surface is then extracted and analyzed in a way like ESI (Hu et al., 2012). ESI on wooden-tip (WT-ESI) was also used for tissue analysis. In this method, a tiny biological sample is loaded on a wooden tip (Hu et al., 2011). When solvent is added and a high voltage is applied to the wooden tip, spray ionization occurs at the wooden tip, and compounds such as proteins, lipids, drugs, and metabolites could be detected from wooden tip-end (Wu et al., 2020; Hu and Yao, 2022).

Recently, there has been an increasing interest for new development and applications of WT-ESI-MS, enabled new applications in various fields such as biologic research, protein study, food safety, forensic investigation, and environmental analysis (Hu and Yao, 2022; Millán-Santiago et al., 2022). In this work, we applied WT-ESI-MS for direct analysis of thyroid tissue samples. Tissue samples adhering to a disposable wooden tip (toothpick) can be sprayed out to generate a characteristic mass spectrum by applying a drop of organic solvent and a high voltage. multivariate data-analytic methods such as principal component analysis (PCA) and partial least squares discriminant analysis (PLS-DA), which are commonly used for biomarker screening in metabolite analysis (Yao et al., 2020; Wu et al., 2021), were applied for biomarker discovery of cancerous thyroid tissues were. Overall, our results show that WT-ESI-MS is a promising tool for tissue analysis and disease diagnosis.

## 2 Materials and methods

### 2.1 Chemicals and materials

**FIGURE 1:**
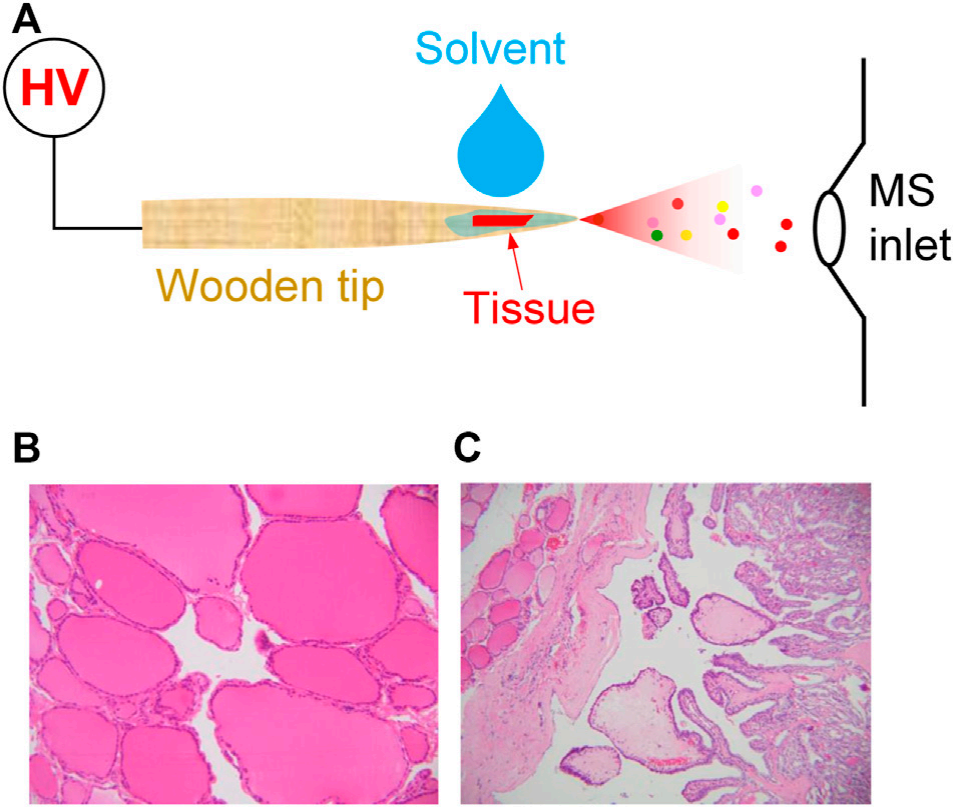
**(A)** WT-ESI-MS analysis of tissue sample; **(B)** normal thyroid tissue, **(C)** thyroid cancer tissue, irregular papillary structure, covered with a layer of neoplastic follicular epithelium with characteristic nuclear changes.

Wooden tips used in this study were common toothpicks purchased from the local supermarket in Guangzhou. The wooden toothpicks are made of natural wood without chemical modification on the surface during manufacturing, as shown in Figure 1A. Before use, wooden toothpicks were washed by methanol and water to clean the surface contaminants. Pure HPLC-grade methanol was purchased from Sigma (St. Louis, USA). All water used in this study is Milli-Q water.

### 2.2 Preparation of clinical samples

Clinical thyroid tissue samples are collected from 23 patients who hospitalized at the Second Affiliated Hospital of Guangzhou University of Chinese Medicine (Guangzhou, China) and have signed informed consents to allow the collection of clinical samples (i.e., cancer thyroid tissue and normal thyroid tissue) for this study. The size of tissue samples at *ca* 1.0 × 1.0 mm. Routine analytical procedures for normal tissue (Figure 1B) and cancer tissue (Figure 1C) were performed by pathological diagnosis of paraffin section to diagnose thyroid cancer.

### 2.3 WT-ESI-MS analysis

To perform wooden-tip MS analysis, the wooden tip was directly mounted on a commercial nanoESI device (Thermo Fisher Scientific, Bremen, Germany) followed previous studies. By placing nanoESI capillary, wooden tip was placed in the front the inlet of mass spectrometer at distances of 1.0 cm horizontal. Pure methanol (5.0 μL), which is commonly used in spray ionization in ambient MS plays a significant role in ambient ESI (McBride et al., 2019), was directly loaded onto wooden tip to extract analytes and generate spray ionization. By applying a high voltage (3.5 kV) onto wooden tip, spray ionization could be generated from wooden tip-end to acquire a characteristic mass spectrum. The capillary temperature was set at 200°C. The high voltage of wooden tip was supplied from the Orbitrap-QE mass spectrometer (Thermo Fisher Scientific, Bremen, Germany). To perform MS/MS experiments, precursor ions were selected with an isolation window at 0.4 Da and a collision energy at 40%.

### 2.4 Ethics

This study was approved by the Ethics Committee of the Second Affiliated Hospital of Guangzhou University of Chinese Medicine (approval number: ZE 2022-371).

### 2.5 Data analysis

All the mass spectral data acquisition and instrumental control were conducted by using Xcalibur 3.0 software (Thermo Fisher Scientific, Bremen, Germany). The acquisition speed was 5.4 scans/sec. Typically, signal duration from the first 1 min were averaged to obtain the high-resolution mass spectra. Principal component analysis (PCA) and partial least squares discriminant analysis (PLS-DA) were conducted on using Simca-p software (Umetrics, Sweden) as described previously (Li W. et al., 2019). Briefly, for MS data of each sample, the intensities of those monoisotopic peaks at the mass range from m/z 700 to 1,100 were normalized. The MS signal intensities higher than 1.0% were input to the software for the PCA and PLS-DA analysis. The variable importance in projection (VIP) value of interest ions that are larger than 1.0 were considered the special biomarkers in this work.

## 3 Results and discussion

### 3.1 Lipid profile of thyroid tissue

**FIGURE 2:**
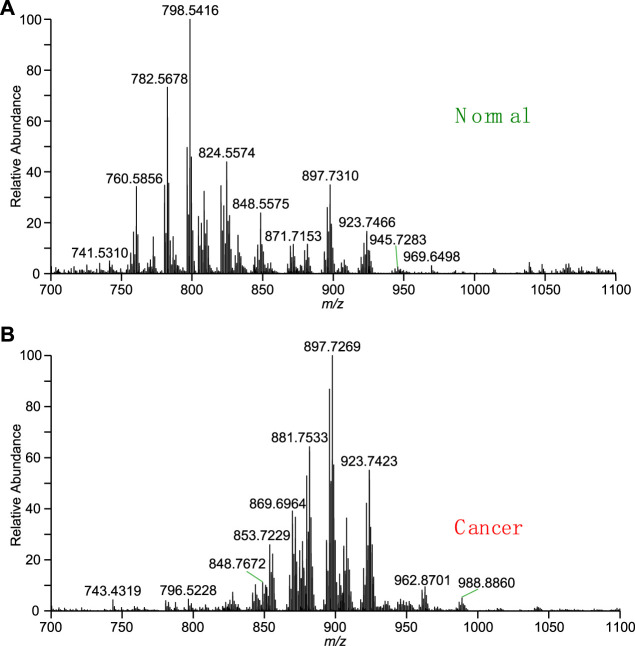
Mass spectra obtained by direct tissue analysis using WT-ESI-MS: **(A)** normal thyroid tissue, **(B)** cancer thyroid tissue.

In order to improve identification of chemical compositions in thyroid tissues, the high-resolution mass spectra of extract of thyroid tissue were observed using WT-ESI-MS in this work. Figure 2A shows mass spectrum of normal thyroid tissue obtained by adding pure methanol. Many peaks were observed from m/z 700 to m/z 1,100 in the mass spectrum where the peak at m/z 782.5678, m/z 798.5416, and m/z 824.5574 are dominated. The base peak at m/z 798.5416 was also used as indicator to evaluate the reproductivity of direct tissue analysis, it was found that the coefficient of variation (CV) of six measurements of normal tissues is 16.3%, which is comparable to WT-ES-MS analysis of other raw complex samples under ambient conditions, showing acceptable reliability for ambient MS analysis (Hu and Yao, 2022). At this mass range, the mass spectrum indicates that there are abundant lipids extracted from of normal thyroid tissue. However, relative abundances of lipids peaks at m/z 897.7269, m/z 881.7533, m/z 923.7423 in the mass spectrum of thyroid cancer tissue were significantly raised, while the relative abundances of lipids such as the peak at m/z 782.5678, m/z 798.5416, and m/z 824.5574 were significantly reduced (Figure 2B). The change of these lipids is believed due to the cancerization of thyroid tissues, and therefore, characteristic spectra of lipid metabolites could be used for diagnosis of disease. It is reported that lipid profile is a type of biomarkers in thyroid cancer (Li D. et al., 2019; Jiang et al., 2021). The study of lipid metabolism of thyroid cancer represents a new field of research that has recently received increasing interest in cancer research. The correlation between lipid profile and cancerization can be changed with metabolic changes. It is reported that a high fatty acid turnover rate is required to offer the energetic and synthetic requirements for the growth of tumor tissue (Maan et al., 2018). In addition, previous studies also reported that lipid profile of patients, including lipid molecules are associated with various types of carcinoma (Tamura et al., 2019). Several metabolic pathways linking it to lipid metabolism are candidate biomarkers to discriminate between normal and cancer cells (Khatami et al., 2019). To the best of our knowledge, only a few studies have investigated the lipid profile of thyroid tissue as a diagnostic tool based ambient MS.

### 3.2 Identification of typical lipids

**FIGURE 3:**
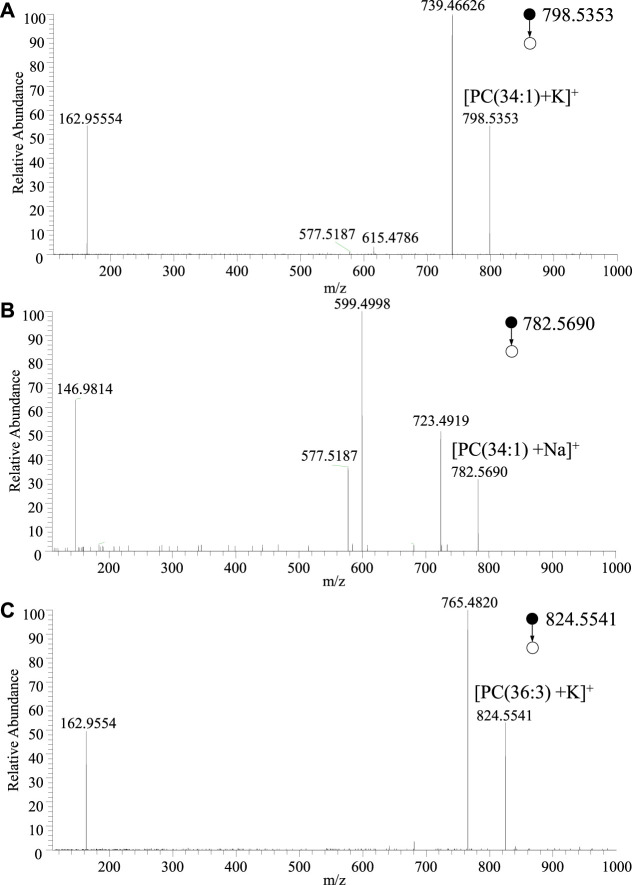
MS/MS spectra of adduct ions of typical lipids obtained by WT-ESI-MS: **(A)** [PC(34:1)+K]^+^, **(B)** [PC(34:1)+Na]^+^, **(C)** [PC(36:3)+K]^+^.

To further validate the typical lipid profile in a large population. The typical lipids at m/z 782.5690, m/z 798.5353, m/z 824.5544, and m/z 913.7456, which are dominated in normal and cancer tissue, respectively, were further investigated. Typical lipids were selected to conduct the MS/MS experiments, as shown in Figure 3. According to the high-resolution mass spectrum, the peak at m/z 798.5416 should be coincided with calculated mass of potassium ions of phosphatidylcholine (PC) [PC(34:1)+K]^+^ (calculated m/z 798.5415) This peak could be assigned to PC adduct within an excellent mass error as low as less than 1 ppm (0.1 ppm). Usually, the mass error less than 5 ppm is excellent for identification of metabolites. For example, upon the collision-induced dissociation (CID), the dissociation of the potassium adducts ([PC(34:1)+K]^+^) led to the detection of different fragment ions in comparison to that of the other adducts. In MS/MS spectrum, the most abundant product ion at m/z 739.4663 is corresponding to the loss of the choline moiety of the head-group (N(CH_3_)_3_) (calculated 59.0735 Da) from the precursor ions, as shown in Figure 3A. A minor peak at m/z 615.4786 relates to the loss of the intact zwitterionic PC head-group (HPO_4_CH_2_CH_2_N(CH_3_)_3_) (calculated 183.0660 Da), and the ions at m/z 577.5187 indicates the further loss of potassium (calculated 38.9637 Da). A fragment ion was detected at m/z 162.9555, which is indicative of the potassium adduct of the phosphate moiety of the head-group ([HPO_4_CH_2_CH_2_+K]^+^) (calculated m/z 162.9563).

Similarly, the peak at m/z 782.5678 should be coincided with sodium adduct of phosphatidylcholine (PC) [PC(34:1)+Na]^+^ (calculated m/z 782.5676). Again, there is an ultra-accuracy mass spectrum with a mass error at 0.25 ppm. Upon MS/MS experiment of [PC(34:1)+Na]^+^, as shown in Figure 3B, the peak at m/z 723.4949 and m/z 599.4998 are corresponding to the natural loss of N(CH_3_)_3_ and HPO_4_CH_2_CH_2_N(CH_3_)_3_ from precursor ions, respectively. The peak at 577.5187 is attribute to the further loss of sodium form m/z 599.4998. The fragment at m/z 146.9814 is generated from the sodium adduct of the phosphate moiety of the head-group ([HPO_4_CH_2_CH_2_+Na]^+^). Although there are different relative abundances of fragment ion form different adducts, the same fragments are very useful for identifying the lipid. Therefore, in the MS/MS spectra of PC ions, the fragment ions at m/z 146.9814 and m/z 162.9554 could indicate the sodium adduct and potassium adduct, respectively, while neutral loss of 59 Da indicates the N(CH_3_)_3_ of PC head group. Therefore, the MS/MS spectrum of the ions at m/z 824.5541 (Figure 3C) could be assigned to the adduct of [PC(36:3)+K]^+^. These identified lipids are good with the lipid database of LIPID MAPS^®^. Owing to high-resolution mass spectrum obtained in this study, chemical compositions of the major peaks of lipids were observed in the averaged mass spectrum were proposed using the accurate mass. According to LIPID MAPS^®^, these results of lipids such as phosphoinositol (PI) and PC are matched in Table 1 with low mass error, which are useful for biomarkers screening of thyroid tissue.

**TABLE 1:** Typical lipids in thyroid tissue. Error (ppm) = [abs (observed mass–calculated mass)/calculated mass]×10^6^.

Lipid adducts	Lipid formula	Observed m/z	Calculated m/z	Error (ppm)
[PC(34:1)+H]+	C42H82NO8P	760.5856	760.5851	0.7
[PC(34:1)+Na]+	C42H82NO8PNa	782.5678	782.5670	1.0
[PC(34:1)+K]+	C42H82NO8PK	798.5416	798.5410	0.8
[PC(36:2)+K+	C44H84NO8PK	824.5574	824.5566	1.0
[PC(38:4)+K]+	C46H84NO8PK	848.5575	848.5566	1.1
[PA 48:1+H]+	C51H99O8P	871.7153	871.7150	0.3
[PA 48:2+H]+	C51H97O8P	869.6964	869.6994	3.4
[PA 50:2+H]+	C53H101O8P	897.7269	897.7307	4.2
[PA 52:3+H]+	C55H103O8P	923.7423	923.7463	4.3

### 3.3 Multivariate variable analysis

**FIGURE 4:**
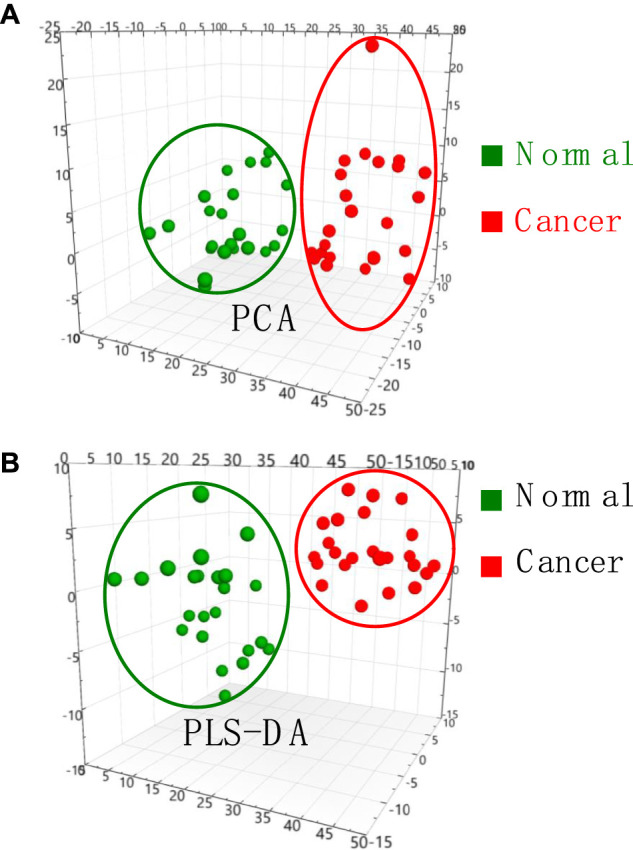
**(A)** PCA analysis and **(B)** PLS-DA analysis of MS data obtained from normal and cancer thyroid tissue samples.

To validate mass spectral fingerprints and specificity of lipid profile, the multivariate variable analysis of MS data was performed to investigate thyroid tissue from different patients. Monitoring change of thyroid tissue would be useful for biomarker discovery. The PCA plot (Figure 4A) show that normal tissue (*n* = 23) and cancer tissue (n = 23) were well separated. In general, it was found that the clusters of normal and cancer tissues are centralized. Although the tissue samples from different individuals, these data indicates that characteristic ions from normal tissue and cancer tissue are significantly dominated due their special lipid metabolites.

To further seek the cancer-induced biomarkers, PLS-DA plots of normal and cancer tissue samples were generated (Figure 4B), showing that normal and cancer thyroid tissues samples are well-separated into two different groups. VIP scores derived from PLS-DA analysis are useful for multivariate analysis for identification of biomarkers. VIP scores >1.0 were considered significant potential candidates of biomarkers, while VIP scores larger than 1.2 are considered for depicting the most significant biomarkers contributing to the cluster group in the PLS-DA model analysis. Therefore, all the most significant biomarkers those VIP scores >1.2 in this work are summarized in Supplementary Table S1. These results showed that lipid profile and potential biomarkers of thyroid cancer can be successfully obtained by WT-ESI-MS, showing a potential tool for rapid diagnosis of thyroid cancer.

## 4 Conclusion

In conclusion, we applied the WT-ESI-MS, a powerful ambient MS technique, to explore lipid profiling of normal and cancer thyroid tissues. The advantage of WT-ESI-MS is that the disposable wooden tip used for sample loading and direct MS analysis of tissue samples without any sample preparation and chromatography separation. Direct MS analysis of each tissue sample can be completed within minutes. In this work, lipids were successfully detected by WT-ESI-MS. Lipids were intensively assigned by high-resolution mass spectrum, and special lipids were also identified by MS/MS. Biomarker discovery was also demonstrated by using PCA and PLS-DA analysis. Overall, our results show that multivariate variable analysis coupled with WT-ESI-MS could be a simple, rapid, and effective analytical tool for investigating the thyroid cancer and other diseases.

## Data Availability

The original contributions presented in the study are included in the article/Supplementary Material, further inquiries can be directed to the corresponding authors.

## References

[B1] Cai S.-H. Di D. Yuan Z.-C. Chen W. Hu B. (2021). Paper-in-Facemask device for direct mass spectrometry analysis of human respiratory aerosols and environmental exposures via wearable continuous-flow adsorptive sampling: A proof-of-concept study. Anal. Chem. 93 (41), 13743–13748. 10.1021/acs.analchem.1c03406 34609849

[B2] Chughtai K. Heeren R. M. A. (2010). Mass spectrometric imaging for biomedical tissue analysis. Chem. Rev. 110 (5), 3237–3277. 10.1021/cr100012c 20423155 PMC2907483

[B3] Griffin J. L. Shockcor J. P. (2004). Metabolic profiles of cancer cells. Nat. Rev. Cancer 4 (7), 551–561. 10.1038/nrc1390 15229480

[B4] Hu B. Lai Y.-H. So P.-K. Chen H. Yao Z.-P. (2012). Direct ionization of biological tissue for mass spectrometric analysis. Analyst 137 (16), 3613–3619. 10.1039/c2an16223g 22574311

[B5] Hu B. So P.-K. Chen H. Yao Z.-P. (2011). Electrospray ionization using wooden tips. Anal. Chem. 83 (21), 8201–8207. 10.1021/ac2017713 21923155

[B6] Hu B. Yao Z.-P. (2018). Detection of native proteins using solid-substrate electrospray ionization mass spectrometry with nonpolar solvents. Anal. Chim. Acta 1004, 51–57. 10.1016/j.aca.2017.11.079 29329708

[B7] Hu B. Yao Z.-P. (2022). Electrospray ionization mass spectrometry with wooden tips: A review. Anal. Chim. Acta 1209, 339136. 10.1016/j.aca.2021.339136 35569859

[B8] Huang L. Mao X. Sun C. Li T. Song X. Li J. (2022). Molecular pathological diagnosis of thyroid tumors using spatially resolved metabolomics. Molecules 27, 1390. 10.3390/molecules27041390 35209182 PMC8876246

[B9] Jiang N. Zhang Z. Chen X. Zhang G. Wang Y. Pan L. (2021). Plasma lipidomics profiling reveals biomarkers for papillary thyroid cancer diagnosis. Front. Cell Dev. Biol. 9, 682269. 10.3389/fcell.2021.682269 34235148 PMC8255691

[B10] Khatami F. Payab M. Sarvari M. Gilany K. Larijani B. Arjmand B. (2019). <p&gt;Oncometabolites as biomarkers in thyroid cancer: A systematic review&lt;/p&gt;. Cancer Manag. Res. 11, 1829–1841. 10.2147/cmar.s188661 30881111 PMC6395057

[B11] Levine A. J. Puzio-Kuter A. M. (2010). The control of the metabolic switch in cancers by oncogenes and tumor suppressor genes. Science 330 (6009), 1340–1344. 10.1126/science.1193494 21127244

[B12] Li D. Zhou L. Ma C. Chen W. Zhang Y. Yu S. (2019a). Comparative analysis of the serum proteome profiles of thyroid cancer: An initial focus on the lipid profile. Oncol. Lett. 18 (3), 3349–3357. 10.3892/ol.2019.10655 31452814 PMC6706610

[B13] Li W. Yao Y.-N. Wu L. Hu B. (2019b). Detection and seasonal variations of huanglongbing disease in navel orange trees using direct ionization mass spectrometry. J. Agric. Food Chem. 67 (8), 2265–2271. 10.1021/acs.jafc.8b06427 30735376

[B14] Maan M. Peters J. M. Dutta M. Patterson A. D. (2018). Lipid metabolism and lipophagy in cancer. Biochem. Biophysical Res. Commun. 504 (3), 582–589. 10.1016/j.bbrc.2018.02.097 PMC6086774 29438712

[B15] Mandal M. K. Yoshimura K. Chen L. C. Yu Z. Nakazawa T. Katoh R. (2012). Application of probe electrospray ionization mass spectrometry (PESI-MS) to clinical diagnosis: Solvent effect on lipid analysis. J. Am. Soc. Mass Spectrom. 23 (11), 2043–2047. 10.1007/s13361-012-0462-3 22923015

[B16] McBride E. M. Mach P. M. Dhummakupt E. S. Dowling S. Carmany D. O. Demond P. S. (2019). Paper spray ionization: Applications and perspectives. TrAC Trends Anal. Chem. 118, 722–730. 10.1016/j.trac.2019.06.028

[B17] Millán-Santiago J. Lucena R. Cárdenas S. (2022). Wooden-based materials: Eco-friendly materials for direct mass spectrometric analysis and microextraction. J. Sep. Sci. 45 (1), 223–232. 10.1002/jssc.202100660 34558202

[B18] Newgard C. B. (2017). Metabolomics and metabolic diseases: Where do we stand? Cell Metab. 25 (1), 43–56. 10.1016/j.cmet.2016.09.018 28094011 PMC5245686

[B19] Nguyen Q. T. Lee E. J. Huang M. G. Park Y. I. Khullar A. Plodkowski R. A. (2015). Diagnosis and treatment of patients with thyroid cancer. Am. Health Drug Benefits 8 (1), 30–40. PMC4415174 25964831

[B20] Nguyen T. M. H. Song W.-Y. Kim T.-Y. (2022). Characterization of spray modes and factors affecting the ionization efficiency of paper spray ionization. Front. Chem. 10, 864184. 10.3389/fchem.2022.864184 35464197 PMC9024139

[B21] Nikiforov Y. E. Nikiforova M. N. (2011). Molecular genetics and diagnosis of thyroid cancer. Nat. Rev. Endocrinol. 7 (10), 569–580. 10.1038/nrendo.2011.142 21878896

[B22] Schneider D. F. Chen H. (2013). New developments in the diagnosis and treatment of thyroid cancer. CA A Cancer J. Clin. 63 (6), 373–394. 10.3322/caac.21195 PMC3800231 23797834

[B23] Shrestha B. Nemes P. Nazarian J. Hathout Y. Hoffman E. P. Vertes A. (2010). Direct analysis of lipids and small metabolites in mouse brain tissue by AP IR-MALDI and reactive LAESI mass spectrometry. Analyst 135 (4), 751–758. 10.1039/b922854c 20349540

[B24] So P.-K. Yang B.-C. Li W. Wu L. Hu B. (2019). Simple fabrication of solid-phase microextraction with surface-coated aluminum foil for enhanced detection of analytes in biological and clinical samples by mass spectrometry. Anal. Chem. 91 (15), 9430–9434. 10.1021/acs.analchem.9b02428 31280558

[B25] Sreekumar A. Poisson L. M. Rajendiran T. M. Khan A. P. Cao Q. Yu J. (2009). Metabolomic profiles delineate potential role for sarcosine in prostate cancer progression. Nature 457 (7231), 910–914. 10.1038/nature07762 19212411 PMC2724746

[B26] Tamura K. Horikawa M. Sato S. Miyake H. Setou M. (2019). Discovery of lipid biomarkers correlated with disease progression in clear cell renal cell carcinoma using desorption electrospray ionization imaging mass spectrometry. Oncotarget 10 (18), 1688–1703. 10.18632/oncotarget.26706 30899441 PMC6422196

[B27] Wang H. Manicke N. E. Yang Q. Zheng L. Shi R. Cooks R. G. (2011). Direct analysis of biological tissue by paper spray mass spectrometry. Anal. Chem. 83 (4), 1197–1201. 10.1021/ac103150a 21247069 PMC3039116

[B28] Wojakowska A. Chekan M. Widlak P. Pietrowska M. (2015). Application of metabolomics in thyroid cancer research. Int. J. Endocrinol. 2015, 258763. 10.1155/2015/258763 25972898 PMC4417976

[B29] Wu L. Yao Y.-N. Yuan Z.-C. Di D. Li L. Hu B. (2020). Direct detection of lysozyme in viscous raw hen egg white binding to sodium dodecyl sulfonate by reactive wooden-tip electrospray ionization mass spectrometry. Anal. Sci. 36 (3), 341–346. 10.2116/analsci.19p288 31656247

[B30] Wu L. Yuan Z.-C. Yang B.-C. Huang Z. Hu B. (2021). *In vivo* solid-phase microextraction swab-mass spectrometry for multidimensional analysis of human saliva. Anal. Chim. Acta 1164, 338510. 10.1016/j.aca.2021.338510 33992222

[B31] Xiao Y. Deng J. Yao Y. Fang L. Yang Y. Luan T. (2020). Recent advances of ambient mass spectrometry imaging for biological tissues: A review. Anal. Chim. Acta 1117, 74–88. 10.1016/j.aca.2020.01.052 32408956

[B32] Yang J. Xiong W. Liu C. Li J. Zhu R. Xia J. (2022). Direct adsorption sampling and ambient mass spectrometry analysis of tobacco smoke with porous paper strips. Front. Chem. 10, 1037542. 10.3389/fchem.2022.1037542 36386000 PMC9643588

[B33] Yao Y.-N. Di D. Yuan Z.-C. Wu L. Hu B. (2020). Schirmer paper noninvasive microsampling for direct mass spectrometry analysis of human tears. Anal. Chem. 92 (9), 6207–6212. 10.1021/acs.analchem.9b05078 32250596

